# Temporal variations in gender identity: an ecological momentary assessment of the influences of context

**DOI:** 10.1080/00049530.2025.2471056

**Published:** 2025-03-03

**Authors:** Karen Man Wa Kwan, Sylvia Yun Shi, Wang Ivy Wong

**Affiliations:** a Department of Applied Social Sciences, Hong Kong Polytechnic University, Hung Hom, Kowloon, Hong Kong; b Gender Studies Programme and Department of Psychology, The Chinese University of Hong Kong, Shatin, New Territories, Hong Kong

**Keywords:** Multidimensional gender identity, ecological momentary assessment (EMA), contextual variations, gender cognitions

## Abstract

**Objective:**

Gender identity is contextually dependent yet is often studied as a static trait. This study used ecological momentary assessment to examine daily variations in multiple dimensions of gender identity (gender salience, gender typicality, gender discontentedness, felt pressure to conform to gender stereotypes) and their associations with gender proportion and location.

**Method:**

Participants (*N* = 138; M_age_ = 19.31; 67 men) completed 4 to 5 reports a day on gender identity and social contexts for 7 days, resulting in 4,409 reports.

**Results:**

All gender identity components showed half as much within-person variance as between-person variance. When the other-gender proportion in the surrounding increased, participants scored higher in gender salience, and men felt more pressure to conform to gender stereotypes than women. When at home (versus other locations), participants scored lower in gender salience, gender typicality, and felt pressure, and men, in particular, reported higher gender discontentedness.

**Conclusions:**

The findings support the social constructivist view that gender identity is dynamic. The findings are discussed in relation to developmental intergroup and distinctiveness theories and social role and reinforcement processes.

## Key Points

**What is already known about this topic:**

1. Social constructivist theories propose that gender identity varies across situations but most studies have treated gender identity as static.
2. Existing research on the contextual variations of gender identity is limited in number and in the choice of gender identity constructs and contexts.
3. Prior research examined how gender identity, defined as masculinity and femininity, changed with different gender composition contexts and locations.

**What this topic adds:**

1. This study investigated daily temporal variations in multiple dimensions of gender identity i.e., gender salience, gender typicality, gender discontentedness, and felt pressure to conform to gender stereotypes using ecological momentary assessment (EMA).
2. Variations in gender proportion were associated with gender salience and felt pressure, while location (home vs. other locations) was related to differences in gender salience, gender typicality, gender discontentedness, and felt pressure.
3. Our findings substantiate the social constructivist theory of gender by shedding light on the fluid and dynamic characteristics of gender identity. Our study also helps to further the study of gender using nontraditional methodology.

Identity and behaviour may differ in public versus in private, with public presentations being more susceptible to social monitoring and in alignment with societal norms. For example, men are more aggressive when under surveillance and women smile more in public (Hyde, 2005). Behaviour may also differ depending on who else is nearby. For example, men’s helping behaviour and cognitive performance are influenced by whether women or men are present (Hyde, 2005; Karremans et al., 2009). Gender is an important social attribute that people use to group others (Martin & Ruble, 2009; Qian et al., 2021; Shutts et al., 2010). It is thus important to study the influence of context on gender identity.

Although the social constructivist view states that gender identity varies with context (Leszczynski & Strough, 2008; Mehta & Dementieva, 2016; Pickard, 2003; Smith et al., 1999), most studies treat them as stable traits by administering one-off measurements. Researchers have highlighted the challenges (e.g., large number of observations, advanced analytical tools) of studying the dynamic changes in gender-related constructs, and have advocated studying their short-term stability and the influence of proximate contextual factors (Martin & Ruble, 2009; Mehta, 2015). As gender identity affects mental well-being (Perry et al., 2019), understanding the contextual nature of gender cognitions may provide insights that could improve well-being. For example, recognising that gender identity is fluid and context-dependent may help challenge biases against gender nonconformity. This understanding can potentially foster more inclusive environments, reducing stigma and enhancing well-being for gender-diverse individuals. Also, as gender identity affects mental well-being (Perry et al., 2019), knowing how it is modified by contextual factors may help identify strategies that potentially improve well-being by moderating certain gender identity components. Few studies have examined contextual variations in gender identity and they have rarely studied such variations naturalistically. Therefore, we repeatedly assessed multiple dimensions of gender identity at random timepoints over a one-week period using ecological momentary assessment (EMA).

First, we introduce the concept of gender identity and review the evidence for and theoretical explanations of contextual variations in gender identity. We then present our hypotheses regarding the effects of two contextual variables, gender proportion and location, on gender identity. We report the results and discuss them in relation to three questions: 1) To what extent does gender identity vary? 2) How does the proportion of gender in the surrounding environment influence gender identity? 3) How does gender identity vary between home and other locations?

## Definitions of gender identity

We focus our review on two approaches that we consider the most relevant for the current study in terms of what has shaped existing studies of variations in gender identity. The traditional approach considers gender identity in terms of gender-typed attributes, namely masculinity and femininity. Studies use the Bem Sex Role Inventory (BSRI) (Bem, 1974), the Personal Attributes Questionnaire (Spence et al., 1975), or inventories derived from these scales (e.g Pickard, 2003)., Masculinity captures agentic qualities e.g., competitiveness and assertiveness whereas femininity captures communal qualities e.g., emotional expressiveness and valuing interpersonal relationships. The concept of masculinity – femininity is associated with meaningful outcomes; for example, higher masculinity predicts greater self-esteem and well-being (Antill & Cunningham, 1980; Martínez-Marín & Martínez, 2019; Wood & Eagly, 2015) and femininity is associated with better relationship quality (Steiner-Pappalardo & Gurung, 2002). However, the concept of masculinity and femininity is confounded with self-endorsed character traits that may change in gender-typing over time (Hoffman & Borders, 2001; Mehta, 2015). Also, research findings on gender differences in masculinity and femininity have been inconsistent. Some studies have reported higher levels of masculinity in men and higher levels of femininity in women based on one-time measures (Oswald, 2004; Özkan & Lajunen, 2005). In contrast, an EMA study found daily variations within individuals but no overall gender differences when using the short version of the BSRI (Mehta & Dementieva, 2016).

A more recent approach considers gender identity as a multi-dimensional construct (Egan & Perry, 2001; Mehta, 2015; Wood & Eagly, 2015). For example, gender identity can be conceptualised as consisting of gender identification (self-identification as a certain gender), gender salience (the degree to which gender is readily noticeable to an individual’s cognitions, particularly in making categorisations) (Bigler & Liben, 2007; Shi et al., 2024), gender typicality (perceived similarity to a gender group), and gender positivity (positive feelings towards a gender group) (Egan & Perry, 2001). This framework resembles an earlier conceptualisation of gender identity as including gender typicality, gender role contentedness, felt pressure to conform to gender stereotypes, and intergroup bias (Egan & Perry, 2001). Measures include self-rating scales (e.g Egan & Perry, 2001; Leaper & Brown, 2008), open-ended questions assessing participants’ tendency to mention gender in self-descriptions (e.g Cota & Dion, 1986; McGuire et al., 1979), and tasks assessing the gender-based playmate preferences and the accuracy of recalling gender-related information (e.g Coyle & Liben, 2016).

Recent research has shown that these gender identity concepts are developmentally meaningful. Low gender typicality, high felt pressure to conform to gender stereotypes, and high gender discontentedness predict negative psychological adjustment such as decreased self-esteem and peer acceptance (Carver et al., 2003; Egan & Perry, 2001; Perry et al., 2019; Yunger et al., 2004), whereas high gender salience may increase gender stereotyping (Hilliard & Liben, 2010; Serbin & Sprafkin, 1986). Men have reported higher gender typicality (DiDonato & Berenbaum, 2013; Skinner et al., 2018; Smith et al., 2018), felt pressure to conform to gender stereotypes, and gender contentedness (Dinella et al., 2014; Egan & Perry, 2001; Smith & Leaper, 2006) than women. For gender salience, men/boys have reported lower gender salience than women/girls in some studies (Pichevin & Hurtig, 1996; Wong et al., 2018), whereas other studies have shown no or mixed gender differences (Cota & Dion, 1986; McGuire & Padawer-Singer, 1976; McGuire et al., 1979; Wong et al., 2018).

## Contextual variations in gender identity

The contextual model of gender, first proposed by Deaux and Major (1987) (see Mehta, 2015 for a review) and consistent with the social constructivist approach, contends that gender identity is contingent upon the situation. The few studies on contextual variations in gender identity have exclusively compared same- versus other-gender contexts (Leszczynski & Strough, 2008; Mehta & Dementieva, 2016; Pickard, 2003; Smith et al., 1999); location, such as private versus public (Echabe & Castro, 1999) or home versus school or workplace (Mehta & Dementieva, 2015); and masculine (e.g., competition, work) versus feminine (e.g., cooperation, home) contexts (Leszczynski & Strough, 2008; Smith et al., 1999). These laboratory-based studies did not directly assess participants’ mental states in real-life contexts. EMA involves collecting data in real life at multiple timepoints to capture the variations in target variables with high ecological validity (Shiffman et al., 2008). Although EMA has been used to measure cognitions, emotions, and behaviours (Mehta, 2015; Shiffman, 2009), only a few studies have used EMA to measure gender-related constructs (Beltz et al., 2021; Mehta & Dementieva, 2015, 2016). In one study, over 90% of the participants showed significant daily variation in self-perceived masculinity and femininity (*d* = 1.55) (Beltz et al., 2021). While prior research highlighted variability in gender identity through masculinity and femininity, no study has yet explored the variability of other gender identity components using a multi-dimensional approach.

### Gender proportion

Studies of how gender identity changes with gender composition have produced mixed findings. Regardless, findings were explained by assuming that people adjust their masculinity or femininity depending on social acceptability. In a laboratory study, both female and male students reported higher femininity after collaborative interaction with other-gender than same-gender partners (Pickard, 2003). The authors drew on social reinforcement theory to explain these findings – men become more feminine when around women because it is more socially acceptable, and women become more feminine when around men because women’s femininity is rewarded in mixed-gender contexts. Adolescent boys and girls both reported higher femininity after playing with girls than with boys (Leszczynski & Strough, 2008). Men reported higher femininity after imagining themselves in an other-gender than a same-gender context, whereas women’s femininity did not vary (Smith et al., 1999).

Overall, studies have suggested that interactions with same- or other-gender persons prompt changes in femininity. However, the contexts in the aforementioned experimental studies may have failed to activate gender schemas related to masculinity (Pickard, 2003). Variations in masculinity may be more apparent in real-life scenarios. It is possible that peer pressure to exhibit masculine behaviours is more pronounced in everyday interactions compared to controlled laboratory settings, which may not fully replicate real daily life. Supporting this possibility, using EMA with BSRI (modified version) in daily life, college men and women reported higher masculinity when with men than with women, although only men reported higher femininity when with women than with men (Mehta & Dementieva, 2016).

The proportion of gender groups may be more relevant than classifying others as exclusively the same versus other-gender or male versus female. Developmental intergroup theory (DIT) (Bigler & Liben, 2007) and distinctiveness theory (Cota & Dion, 1986; McGuire, 1984) suggest that a social category becomes more salient when it represents the minority group, leading to increased enactment of cognitions and behaviours typical of that categorical identity. Supporting this hypothesis, experimental (Cota & Dion, 1986) and cross-sectional (McGuire et al., 1979) studies have shown that people feel more gender salient when they are the minority gender in a mixed-gender group than when they are the majority gender or in a single-gender group. Therefore, DIT and distinctiveness theory predict changes in gender identity as the proportion of other-gender increase, e.g., man become more gender-salient, more masculine or gender-typical in the presence of more women. In contrast, the social reinforcement theory suggests that men become more feminine or less gender-typical in the presence of more women, because it is more socially acceptable in mixed-gender contexts, and prior research has reported such findings (Pickard, 2003). Further research is needed to examine these competing hypotheses and the effects on other dimensions of gender identity, i.e., gender contentedness and felt pressure to conform to gender stereotypes.

### Location

Another focus of research on contextual variations in gender is that of location, specifically private versus public contexts. In a laboratory study, men reported higher femininity when they imagined being at home than being at school or at work, while both men and women reported higher masculinity at work than in other locations (Smith et al., 1999). Similarly, an unpublished ecological study (Mehta & Dementieva, 2015) showed that both men and women reported higher femininity when at home than in other locations (i.e., school, workplace). These variations in masculinity and femininity may reflect individuals’ different goals in private versus public contexts (Echabe & Castro, 1999). In private contexts (e.g., home), where maintaining interpersonal relationships is an important goal, people may display more feminine (i.e., communal) characteristics; in public contexts (e.g., work, school), performance is an important goal, people may display more masculine (i.e., agentic) characteristics. However, no study has tested how location influences gender identity components beyond masculinity and femininity.

Location may be theorised to affect gender salience, gender typicality, felt pressure to conform to gender stereotypes, and gender discontentedness. Individuals are socialised to conform to expected gender norms through interactions with parents, peers, and teachers. Social role expectations become internalised during self-socialisation, leading to self-monitoring and adherence to gender norms (Leaper & Friedman, 2007). Individuals may modify their behaviours when they are being observed or identified. Individuation can lead to increased gender-typing of behaviours and stronger self-perceptions (Hyde, 2005). People experience societal pressure to conform to gender roles (Eagly & Wood, 2016; Hyde, 2005), leading to more gendered behaviours in public. Public places also present more opportunities for people to experience negative gender-related events, such as body image judgements from friends and strangers (Wasylkiw & Williamson, 2013) and gender-discrimination in the workplace (Bobbitt-Zeher, 2011). Thus, being at home or alone – where there is less individuation, less social pressure, and fewer sources of gender discontent – may be associated with lower levels of gender salience, gender typicality, felt pressure to conform to gender stereotypes, and gender discontentedness.

## The present study

While previous studies have compared masculinity and femininity in single-gender contexts, the present study used EMA to examine variations in gender identity dimensions (i.e., gender salience, gender typicality, gender discontentedness, and felt pressure to conform to gender stereotypes) in everyday life. We examined gender proportion and location (home versus other locations) as contextual dimensions. We tested three hypotheses:

H_1_: **Temporal Variability Across Assessments**. We investigated variations in gender identity across the study period. Based on the contextual model (Deaux & Major, 1987) and social constructivist approach (Leszczynski & Strough, 2008; Mehta & Dementieva, 2016; Pickard, 2003; Smith et al., 1999), as well as two EMA studies on masculinity and femininity (Beltz et al., 2021; Mehta & Dementieva, 2016), we hypothesised that gender salience, gender typicality, felt pressure to conform to gender stereotypes, and gender discontentedness would show daily variations in everyday life (H_1_).

H_2_: **Gender Proportion**. We drew on DIT (Bigler & Liben, 2007) and distinctiveness theory (Cota & Dion, 1986; McGuire, 1984) to formulate our predictions about gender proportion, as they offer clearer and more systematic set of hypotheses than social reinforcement theory (Pickard, 2003) or social role theory (Eagly & Wood, 2016; Hyde, 2005). When the proportion of other-gender increases such that one gender becomes the minority group, their own gender identity and gender-typical features become more salient. Salience has been theorised to predict more gender-conforming cognitions, which may encompass felt gender typicality and felt pressure to conform. Studies also found that felt gender typicality is positively correlated with gender contentedness (Carver et al., 2003; Dinella et al., 2014; Egan & Perry, 2001; Smith & Leaper, 2006). Therefore, we hypothesised that with a higher proportion of other-gender, participants would report higher gender salience (H_2a_), higher gender typicality (H_2b_), higher felt pressure to conform to gender stereotypes (H_2c_), and lower gender discontentedness (H_2d_).

H_3_: **Location**. Because in private (compared to in public), there is less consideration of social expectations (Eagly & Wood, 2016; Hyde, 2005), less individuation and thus less gender-typing and weaker self-perceptions (Hyde, 2005), and fewer opportunities to elicit gender discontent (Bobbitt-Zeher, 2011; Wasylkiw & Williamson, 2013), we expected participants to report lower gender salience (H_3a_), gender typicality (H_3b_), felt pressure to conform to gender stereotypes (H_3c_), and gender discontentedness (H_3d_), when at home than in other locations.

Our sample consisted of Chinese young adults in Hong Kong. Our results are expected to be generalisable to similar populations in developed societies because studies using industrialised Chinese and Western samples have reported similar findings for gender cognition development and social influences (Gibbons, 2000; Lobel et al., 2000; Qian et al., 2021; Wong & VanderLaan, 2020). However, the inclusion of a Chinese sample diversifies psychological research, which has relied heavily on Euro-American samples (Nielsen et al., 2017).

## Method

### Participants

Participants were recruited through the participant pool of a psychology course, posters on campus, and mass emails sent to students at a university in Hong Kong. Prior EMA studies on gender cognitions used small samples of youth and young adults, with reports ranging from hundreds to thousands (Beltz et al., 2021, *N*_*participants*_ = 57, *N*_*reports*_ >4000; Mehta & Dementieva, 2016, *N*_*participants*_ = 27, *N*_*reports*_ = 448; Mehta et al., 2016, *N*_*participants*_ = 20, *N*_*reports*_ = 2211). This study recruited an initial sample of 179 participants. After screening out those who did not complete the whole study, the final sample included 138 participants (67 men and 71 women; *M*_*Age*_ = 19.32, *SD*_*Age*_ = 1.54). See Table 1 for demographic characteristics.

**Table 1.** Participants’ demographic characteristics (*N* = 138).

Variables	Range	Frequency (%) or Mean (SD)
Sex^a^	NA	Men: 67 (48.6%); Women: 71 (51.4%)
Age	17–26	19.31 (1.53)
Field of study	NA	Arts and Humanities and Social Science: 84 (60.9%) Science and Engineering: 54 (39.1%)
Sexual orientation^a^	NA	Exclusively heterosexual: 92 (66.7%) Predominantly heterosexual, only incidentally homosexual: 12 (8.7%) Predominantly heterosexual, but more than incidentally homosexual: 6 (4.3%) Equally heterosexual and homosexual: 13 (9.4%) Predominantly homosexual, but more than incidentally heterosexual: 4 (2.9%) Predominantly homosexual, incidentally heterosexual: 6 (4.3%) Exclusively homosexual: 5 (3.6%)
Education level of father	NA	Primary school and below: 8 (5.8%) Junior secondary school: 20 (14.5%) Senior secondary school: 42 (30.4%) Associate degree/Higher diploma: 5 (3.6%) Bachelor’s degree: 37 (26.8%) Master’s degree and above: 26 (18.8%)
Education level of mother	NA	Primary school and below: 7 (5.1%) Junior secondary school: 12 (8.7%) Senior secondary school: 53 (38.4%) Associate degree/Higher diploma: 18 (13.0%) Bachelor’s degree: 30 (21.7%) Master’s degree and above: 18 (13.0%)
Citizenship	NA	Hong Kong: 124 (89.9%); Mainland China: 5 (3.6%); Taiwan: 1 (0.7%); Korea: 2 (1.4%); India: 1 (0.7%); Malaysia: 1 (0.7%); Australian: 1 (0.7%) British: 1 (0.7%) USA: 2 (1.4%)
Religion	NA	No religion: 96 (69.6%); Christian: 31 (22.5%); Catholic: 4 (2.9%); Protestant: 2 (1.4%); Buddhist: 2 (1.4%); Muslim: 1 (0.7%); Others: 2 (1.4%)

^a^Sex, sexual orientation and religion were based on participants’ self-identification. The sexual orientation question was based on the Kinsey scale (Kinsey et al., 1948).

### Procedures

Ethical approval was obtained from the University of Hong Kong. In a briefing session, the participants gave consent, completed demographic questionnaire, and installed mobile app (Paco) on their phones (running iOS or Android). After the briefing, each participant completed an EMA survey four to five times daily for seven days. The app sent five random signals within a 12-hour window set by the participant (e.g., 10 am to 10pm) to prompt the survey, which took about 2.5 minutes. If the survey was not completed, a reminder popped up after 15 minutes, which disappeared if there was still no response after 15 minutes. Participants were allowed to initiate one “make-up” survey per day if they missed any signal. Participants were required to complete a minimum of 4 out of 5 surveys per day for 7 days. By the end of the study, a total of 4,409 out of a possible 4,820 reports were submitted, representing 91% of the maximum possible reports. Also, 100% of participants met the requirement of completing at least 4 reports daily. After completion of the whole study, participants who were recruited from the participant pool received course credits and the others received ~USD13 in compensation.

### EMA scale measures

To reduce fatigue and boredom from multiple assessments, we focused on variables susceptible to contextual influences and created short measures, as recommended by previous EMA research (Mehta & Dementieva, 2016; Mehta et al., 2016; Solhan et al., 2009; Sunner et al., 2013). Each measure of gender identity (described below) included three items adapted from trait measures by adjusting the wording to reflect momentary gender identity in adults. For example, the original item of felt pressure to conform to gender stereotypes for one-time measure in adolescents, ‘‘My parents would be upset if I wanted to learn an activity that only boys usually do”, was adapted to “Right now, I think others will be upset if I want to learn an activity that only men usually do”. Pilot studies were conducted to test the scales’ reliabilities. In the first pilot study, the draft EMA questionnaire was distributed to 100 university students. Pilot participants completed the questionnaire only once. The Cronbach’s *α* values of reliabilities in the sample were ≥ 0.70 for all except two scales: felt pressure to conform to gender stereotypes and gender discontentedness. In a second pilot study, these two scales were revised to improve readability and three additional items were adapted from prior research. Seventy-four university students completed the second pilot study. The three items that contributed to the highest reliability for felt pressure to conform to gender stereotypes (Cronbach’s α = 0.82) and gender discontentedness (Cronbach’s α = 0.74), respectively, were adopted for the main study. The term “sex” was used in the demographic questionnaire to ask participants to indicate their (biological) sex, with no participant indicating a transgender or non-binary identity. The term “gender” was used throughout the EMA questionnaire because it measured various aspects of gender identity, and is thus used throughout the paper. All items were measured on 7-point scales from “Not at all” to “Very much/A lot”. There were separate versions of each item for men and women, and they completed their respective versions.

#### Gender salience

Three items adapted from Palomares (2009) assessed momentary gender salience: “Right before taking this questionnaire, how much were you aware of being a man/woman?” “Right before taking this questionnaire, to what extent were you paying attention to others’ gender?” “How much do you think gender influenced you in the situation right before taking this questionnaire?” Item scores were averaged, and higher scores indicated higher gender salience (Cronbach’s α = 0.79).

#### Felt gender typicality

Leaper and Brown’s (2008) multi-dimensional gender identity scale was adapted to form a three-item scale of momentary felt gender typicality: “Right now, I feel just like men/women of my age”. “Right now, I feel I fit in with other men/women”. “Right now, I think I am a good example of men/women”. Item scores were averaged, and higher scores indicated higher felt gender typicality (Cronbach’s α = 0.87).

#### Felt pressure to conform to gender stereotypes

Three items adapted from Leaper and Brown (2008) assessed felt pressure to conform to gender stereotypes: “Right now, I think others will not like me if I do things that men/women usually do”. “Right now, I think others will be upset if I want to learn an activity that only men/women usually do”. “Right now, I think others will mind if I don’t act like a woman/man”. Item scores were averaged, and higher scores indicated higher felt pressure (Cronbach’s α = 0.92).

#### Gender discontentedness

Three items, adapted from Leaper and Brown (2008), assessed momentary gender discontentedness: “Right now, how much do you think it would be better if you were a man/woman?” “Right now, I like being a man/woman”. “Right now, I feel happy to be a man/woman”. The second and third items were reverse coded so that higher scores represented higher gender discontentedness. Item scores were averaged (Cronbach’s α = 0.69).

### Context measures

#### Other-gender proportion

The participants were asked to estimate “How many males/females are present around you right now?” The other-gender proportion was calculated by dividing the number of other-gender persons (persons of all genders other than the participant’s own) by the total number of persons reported (including the participant). Despite acknowledging the potential presence of transgender and nonbinary people, participants were not asked to estimate their presence due to their low visibility in Chinese societies and the need to keep the scales brief.

#### Location (home versus other)

The participants reported their current location by choosing one of the following options: home, school, transport, friend’s home, and other. If they chose “other”, they had to type a response. Home was considered a private context and other locations were considered public contexts.

### Statistical analyses

We first investigated the daily variations in gender identity to test the hypothesis regarding the daily variations in gender identity (H_1_). The daily variations were estimated for each person by calculating the intraindividual standard deviations (iSDs) of each gender identity component across all time points over the seven days, following the analytic approach of Beltz et al. (2021).

Then, we investigated the contextual variations in gender identity (H_2_ and H_3_). Before conducting the main analyses, we examined all the demographic variables (i.e., sex, age, field of study, sexual orientation, education level of parents, citizenship, and religion) to test for possible confounds. For the field of study, participants were asked an open-ended question. The responses were then coded into either “Arts and Humanities and Social Sciences” or “Science and Engineering”, which are broadly recognised as showing gender differences (Speer, 2017; Trusz, 2020). Gender differences in age, sexual orientation, and parents’ education level were examined using t-tests while gender differences in field of study, citizenship, and religion were examined using chi-square tests. Also, correlation analyses were conducted between all the demographic variables and dependent variables (i.e., gender salience, gender typicality, gender discontentedness, and felt pressure to conform to gender stereotypes). Variables that differed significantly for men and women and correlated with any of the dependent variables were controlled for in the main analyses. A significant gender difference was found in age, with men being older than women, and in field of study, with a higher proportion of women in arts and humanities and social science, and a higher proportion of men in science and engineering. Additionally, age and field of study were correlated with most dependent variables. Thus, age and field of study were controlled for in the main analyses.

The main analyses were based on 4,409 EMA reports from the 138 participants (each participant contributed 28 to 35 reports). We tested the effects of other-gender proportion and location on each gender identity measure. The percentages of reports completed in each location were: home (49.6%), school (22.7%), transport (9.3%), friend’s home (1.5%), and other (16.9%). Given our aim to compare private and public contexts, all responses other than home (private context) were combined into other locations (public context, 50.4%) for parsimony. As the participants were more likely to be alone at home than in other locations, gender proportion and location were entered simultaneously as predictors to distinguish their unique effects (i.e., to avoid confounding location effects from the effects of being alone).

In the main analyses, general linear mixed effects models were conducted. Repeated measures were nested within participants. Fixed effects included other-gender proportion, location (home versus other), participant gender, and interaction terms (gender × other-gender proportion, gender × location). Participants were treated as a random effect. Separate models were conducted for each dependent variable (i.e., gender salience, gender typicality, gender discontentedness, and felt pressure to conform to gender stereotypes). Age and field of study were entered as covariates. We also reported gender differences in the gender identity variables.

## Results

### Daily variations in gender identity (H_1_)

**Figure 1.**
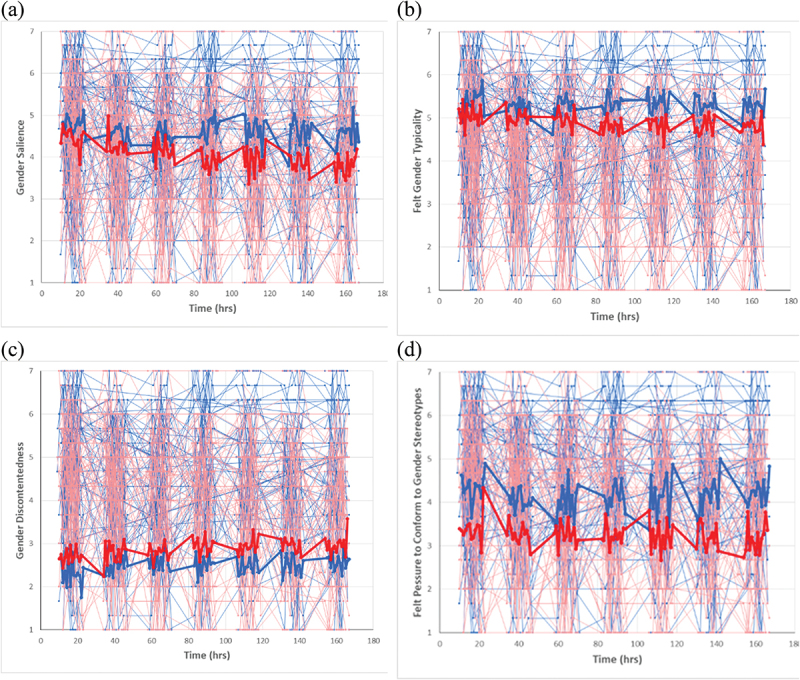
Daily variations in gender identity for individual men (thin blue lines) and women (thin red lines) over the 7 days. One line represents one participant. Thick blue lines show the means for men and thick red lines show the means for women. Time points with fewer than 10 data points were excluded. On the x-axis, “0 hr” refers to the time point 0:00 on the first day of the experiment. For example, a random signal emitted at 10 am on the first day would correspond to “10 hrs” on the x-axis.

Table 2 presents the iSD analyses and Figure 1 illustrates the daily variations in gender identity. Each iSD score reflects the variance in an individual’s score relative to their own mean; therefore, larger iSDs reflect greater intra-person variability and an iSD of zero indicates no variability. One-sample *t*-tests were used to examine whether the average sample iSDs significantly differed from zero. The mean iSDs for all gender identity components were greater than zero, indicating substantial variation, *p*s < .001, *d*s >1.00. Independent samples *t*-tests were conducted to examine gender differences in the variations in the iSDs. No significant gender difference was found for the iSDs of any gender identity component, suggesting that men’s and women’s gender identity fluctuated to a similar extent.

**Table 2.** Variations in gender identity using iSds.

	Min *iSD*	Max *iSD*	Mean *iSD*	*SD iSD*	One-sample *t*-test of *iSDs (reference point: 0)*	Participants with significant variations (*iSD* > 0)	Independent samples *t*-test for gender difference in *iSD*	Mean_iSD_/*SD*_M_
Gender salience	0	8.60	.81	.79	*t*(137) = 12.10, *p* < .001, *d* = 1.03	98.55%	*t*(136) = .12, *ns*	.53
Felt gender typicality	0	1.49	.49	.29	*t*(137) = 19.97, *p* < .001, *d* = 1.70	92.75%	*t*(136) = −.41, *ns*	.43
Gender discontentedness	0	1.50	.45	.26	*t*(137) = 20.26, *p* < .001, *d* = 1.72	94.93%	*t*(136) = 1.81, *ns*	.42
Felt pressure to conform to gender stereotypes	0	2.81	.73	.42	*t*(137) = 20.25, *p* < .001, *d* = 1.72	97.83%	*t*(136) = −.52, *ns*	.45

*iSD* = intraindividual standard deviation, Min *iSD* = minimum value of *iSD*. Max *iSD* = maximum value of *iSD*, Mean *iSD* = mean value of *iSD*, *SD iSD* = overall sample *SD* of *iSD*, *SD*_*M*_ = *SD* of mean gender identity.

Next, the within- and between-person variations were compared. For each gender identity component, the mean iSD across participants was divided by the SD of the mean gender identity score for all time points (i.e., M_iSD_/SD_M_). The results in Table 2 show that the within-person variance was around half as large as the between-person variance: M_iSD_/SD_M_ ranged from .42 to .53. This indicates that the variability in gender identity within individuals over time (within-person variance) is about half the variability observed between different individuals (between-person variance). Across individuals, the gender identity iSDs ranged from 0 to 8.6, with 92.75% to 98.55% of the participants reporting statistically significant variations (iSDs >0, all *ds* > 1) for each gender identity component. M_iSD_s represent the average within-person variation across all participants. The larger M_iSD_s values for gender salience (0.81) and felt pressure to conform to gender stereotypes (0.73) indicate greater fluctuation, which may be influenced by environmental factors. In contrast, the smaller M_iSD_s values for felt gender typicality (0.49) and gender discontentedness (0.45) suggest these constructs are more stable across different situations.

### Contextual variations in gender identity (H_2_ and H_3_)

The results regarding the effects of other-gender proportion and location on each gender identity measure are reported below and in Table 3:

**Table 3.** Results of general linear mixed effects models.

	Main Effects			Interactions	
Gender Identity Variables	Other-gender Proportion	Location	Gender	Gender × Other-gender Proportion	Gender × Location
Gender salience	***F*****(1, 4306) = 26.44, *p* < .001, *β* = 0.27**	***F*****(1, 4297) = 64.61, *p* < .001, *d* = .16**, $\overset{ˉ}{X}$_**home**_** = 4.23**, $\overset{ˉ}{X}$_**other locations**_** = 4.47**	***F*****(1, 141) = 7.30, *p* = .008, *d* = .43**, $\overset{ˉ}{X}$_**Male**_** = 4.68**, $\overset{ˉ}{X}$_**Female**_** = 4.02**	*F*(1, 4306) = 1.13, *n.s.*	*F*(1, 4297) = .12, *n.s.*
Gender typicality	*F*(1, 4298) = 2.87, *n.s.*	***F*****(1, 4291) = 21.42, *p* < .001, *d* = .08**, $\overset{ˉ}{X}$_**home**_** = 5.07**, $\overset{ˉ}{X}$_**other locations**_** = 5.16**	***F*****(1, 141) = 9.16, *p* = .003, *d* = .49**, $\overset{ˉ}{X}$_**Male**_** = 5.39**, $\overset{ˉ}{X}$_**Female**_** = 4.84**	*F*(1, 4298) = .25, *n.s.*	*F*(1, 4291) = .018, *n.s.*
Gender discontentedness	*F*(1, 4297) = 3.13, *n.s.*	*F*(1, 4290) = 1.05, *n.s.*	***F*****(1, 140) = 11.29, *p* = .001, *d* = −.59, $\overset{ˉ}{X}$**_**Male**_** = 2.42**, $\overset{ˉ}{X}$_**Female**_** = 2.96.**	*F*(1, 4297) = 2.03, *n.s.*	***F*****(1, 4290) = 4.11, *p* = .043** Posthoc: Men reported higher gender discontentedness at home than in other locations, *F*(1, 2071) = 5.50, *p* = .019, *d* = −.05, $\overset{ˉ}{X}$_home_ = 2.48, $\overset{ˉ}{X}$_other locations_ = 2.43. N.S. for women, *F*(1, 2219) = .42, *n.s.*
Felt pressure to conform to gender stereotypes	***F*****(1, 4301) = 15.29, *p* < .001, *β* = .12**	***F*****(1, 4293) = 74.82, *p* < .001, *d* = .16**, $\overset{ˉ}{X}$_**home**_** = 3.58**, $\overset{ˉ}{X}$_**other locations**_** = 3.84**	***F*****(1, 141) = 10.87, *p* = .001, *d* = .53, $\overset{ˉ}{X}$**_**Male**_** = 4.14**, $\overset{ˉ}{X}$_**Female**_** = 3.27**	***F*****(1, 4301) = 4.65, *p* = .031** Posthoc: When other-gender proportion increased, men reported higher pressure to conform to gender stereotypes, *F*(1, 2078) = 18.92, *p* < .001, *β* = .40. N.S. for women, *F*(1, 2223) = 1.50, *n.s.*	*F*(1, 4293) =.069, *n.s.*

Significant effects are bolded, and the means are the estimated marginal means adjusted by the model.

#### Context and gender salience

**Figure 2.**
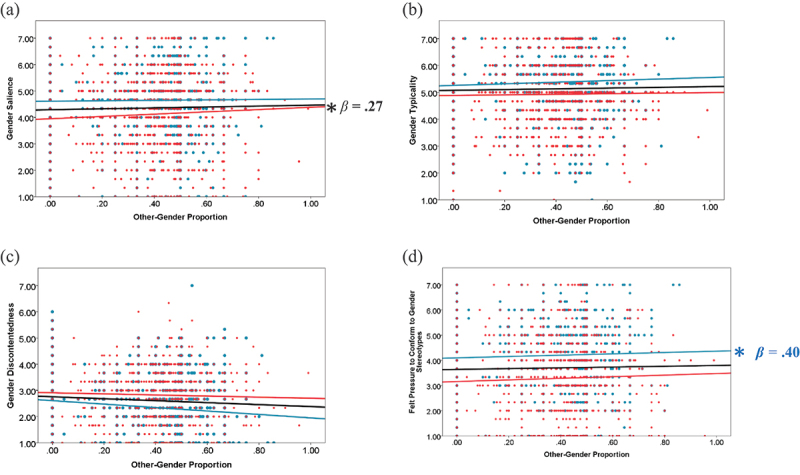
Scatter plots showing the variations in gender identity with other-gender proportion for men (blue dots) and women (red dots). Blue lines are the fit lines for men, red lines are the fit lines for women, black lines are the fit lines for all participants. *p < .05.

**Figure 3.**
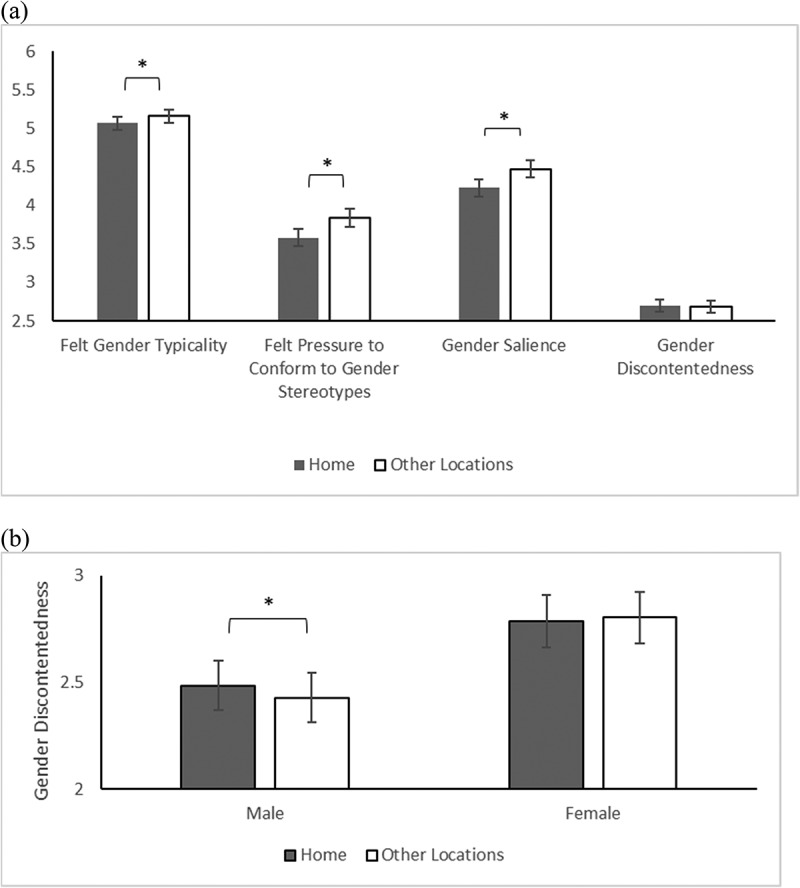
Panel (a) shows main effects of location for each gender identity component; panel (b) shows the gender × location interaction for gender discontentedness. *p < .05.

The hypothesis (H_2a_) that other-gender proportion would increase gender salience was supported, *F*(1, 4306) = 26.44, *p* < .001, *β* = 0.27 (see Figure 2a). The hypothesis (H_3a_), which predicted lower gender salience at home than in other locations, was also supported, *F*(1, 4297) = 64.61, *p* < .001, *d* = .16, $\overset{ˉ}{X}$_home_ = 4.23, $\overset{ˉ}{X}$_other locations_ = 4.47 (see Figure 3a). The main effect of gender showed that men reported higher gender salience than women, *F*(1, 141) = 7.30, *p* = .008, *d* = .43, $\overset{ˉ}{X}$_Male_ = 4.68, $\overset{ˉ}{X}$_Female_ = 4.02. Gender did not significantly interact with context.

#### Context and gender typicality

Gender typicality did not increase with other-gender proportion, *F*(1, 4298.47) = 2.87, *n.s*. (see Figure 2b). Thus, the hypothesis (H_2b_) was not supported. The hypothesis (H_3b_) that gender typicality would be lower at home than in other locations was supported, *F*(1, 4291.10) = 21.42, *p* < .001, *d* = .08, $\overset{ˉ}{X}$_home_ = 5.07, $\overset{ˉ}{X}$_other locations_ = 5.16 (see Figure 3a). The main effect of gender showed that men reported higher gender typicality than women, *F*(1, 141) = 9.16, *p* =.003, *d* = .49, $\overset{ˉ}{X}$_Male_ = 5.39, $\overset{ˉ}{X}$_Female_ = 4.84. Gender did not significantly interact with context.

#### Context and felt pressure to conform to gender stereotypes

The hypothesis (H_2c_) that as other-gender proportion increased, participants would feel higher pressure to conform to gender stereotypes was partially supported by a significant interaction between gender and other-gender proportion, *F*(1, 4301) = 4.65, *p* = .031. Follow-up analyses by gender showed that the effect of gender proportion was significant for men, *F*(1, 2078) = 18.92, *p* < .001, *β* = .40, but not for women, *F*(1, 2223) = 1.50, *n.s*.(Figure 2c). The hypothesis (H_3c_) that participants felt less pressure to conform to gender stereotypes at home than in other locations was also supported, *F*(1, 4293) = 74.82, *p* < .001, *d* = .16, $\overset{ˉ}{X}$_home_ = 3.58, $\overset{ˉ}{X}$_other locations_ = 3.84 (see Figure 3a). The main effect of gender showed that men felt higher pressure than women, *F*(1, 141) = 10.87, *p* = .001, *d* = .53, $\overset{ˉ}{X}$_Male_ = 4.14, $\overset{ˉ}{X}$_Female_ = 3.27.

#### Context and gender discontentedness

The hypothesis (H_2d_) that gender discontentedness would be higher when other-gender proportion increased was not supported, *F*(1, 4297.13) = 3.13, *n.s*. (see Figure 2d). The hypothesis (H_3d_) that participants would be less discontented at home than in other locations was not supported, *F*(1, 4290.09) = 1.05, *n.s*. (see Figure 3a). However, there was a significant interaction between gender and location, *F* (1, 4290) = 4.11, *p* = .043. Follow-up analyses by gender showed that men reported higher gender discontentedness at home than in other locations, *F*(1, 2071) = 5.50, *p* = .019, *d* = −.05, $\overset{ˉ}{X}$_home_ = 2.48, $\overset{ˉ}{X}$_other locations_ = 2.43, whereas there was no significant difference for women, *F*(1, 2219) = .42, *n.s*. (see Figure 3b). The main effect of gender showed that women reported greater discontentedness than men, *F*(1, 140) = 11.29, *p* = .001, *d* = −.59, $\overset{ˉ}{X}$_Male_ = 2.42, $\overset{ˉ}{X}$_Female_ = 2.96.

### Additional analysis

In our main analyses, other-gender proportion was treated as a continuous variable to preserve variability information. However, this approach did not separate the effects of the participants being alone versus being with exclusively same-gender persons, as both scenarios were coded as 0.

To address this potential limitation on the interpretation of findings concerning gender proportion, we converted the other-gender proportion into a categorical variable. Because the datapoints showed a high frequency of being alone (40.5%) and low frequency of being in a predominantly other-gender environment (7.5%) (see Table S1), we recoded the data into four categories: alone = 0, other-gender proportion smaller than .50 (self as majority gender) = 1, equal proportion of other-gender (self-gender equally represented) = 2, and proportion of other-gender larger than .50 (self as minority gender) = 3. For details, refer to the additional analysis shown in the supplementary materials.

In sum, participants reported lower gender salience, typicality, and pressure to conform to gender stereotypes when alone than when with others, irrespective of the group’s gender proportion. These findings are consistent with established findings and with socialisation and social role theories (Hyde, 2005; Leaper & Friedman, 2007). That is, the presence of others, regardless of their gender, heightens gender awareness and imposes gender socialisation pressure.

In contrast, in the additional analysis, when including the effect of “being alone”, gender proportion only increased male participants’ gender discontentedness when they were in a female-majority group, not when they were in a male-majority group or alone. The weakened support for the gender proportion effect is likely attributable to the loss of data variability when converting the other-gender proportion from a continuous to a categorical variable. The main analysis that treated gender proportion as a continuous variable and included location as a simultaneous predictor, was appropriate for several reasons. First, although considering other-gender proportion as a continuous variable did not differentiate between being alone and in a same-gender group, the purpose of differentiation was to account for differences in privacy and social monitoring. Including location in main analysis meant that the extent of privacy and social monitoring was controlled to some extent when the effect of gender proportion was being tested. Second, datapoints representing other-gender proportions equal to (10.6%) or larger than .50 (7.5%) were insufficient (see Table S1). This limited the number of categories that could be created to represent the different gender proportion groups and thus lowers the sensitivity of the categorical analysis. Third, excluding cases where the participants were alone in the main analysis while keeping gender proportion as a continuous variable may not be a good option because this would entail removing half of the datapoints.

## Discussion

People’s self-presentation varies by situation, with gender significantly influencing how people act, feel, and think (Bigler & Liben, 2006; Hyde, 2005; Maccoby, 2000; Martin & Ruble, 2009). While most studies consider gender identity static, social constructivist theories propose that it varies across situations (Cook et al., 2022; Deaux & Major, 1987; Echabe & Castro, 1999; Hyde, 2005; Martin & Ruble, 2009; Mehta, 2015; Wood & Eagly, 2015). We examined multiple components of gender identity in daily life using an ecological momentary method. Our findings showed that gender identity varied depending on gender composition and location.

### Does gender identity fluctuate across time?

We found substantial variations in all gender identity components over the one-week study period, supporting H_1_. Between 92.75% and 98.55% of the participants reported variations in each gender identity component. The within-person variance equated to approximately half of the between-person variance. Beltz et al. (2021) similarly showed substantial daily variation in 93% of young adult participants’ self-rated gender self-concept. The within-person variance was around a third of the between-person variance. These convergent results demonstrate daily variations in gender identity within individuals. Mehta and Dementieva (2016) similarly reported within-person variations in masculinity and femininity, although they did not perform an iSD analysis. By using a multidimensional measure of gender identity, our study extends previous EMA research (Beltz et al., 2021; Mehta & Dementieva, 2016) and experimental studies on the influence of context on gender identity (Leszczynski & Strough, 2008; Pickard, 2003; Smith et al., 1999). Our Chinese sample provides cross-cultural validation of these findings. Together, the findings of several studies support the social constructivist hypothesis that gender identity is malleable over time.

### How does gender identity vary with gender proportion?

Our findings demonstrate that gender composition in daily contexts is associated with gender identity components other than masculinity and femininity. The results supported the hypothesis that a higher proportion of other-gender is associated with higher gender salience (H_2a_), based on the reasoning that group-specific traits become more distinctive for those in a minority and serve as a basis for self-categorisation (Bigler & Liben, 2007; Cota & Dion, 1986; McGuire, 1984).

Felt pressure to conform to gender stereotypes was also associated with a higher proportion of other-gender, although only among men, partially supporting H_2c_. This finding is consistent with other studies that have found that gender norms are regarded as more serious for men than women, and gender-nonconformity in men is judged more negatively than women by peers (Kwan et al., 2020; Wallien et al., 2010). The high pressure on men to conform to gender stereotypes when more women are present might be explained by DIT and distinctiveness theory as well as social role and reinforcement processes. That is, a female-dominated context may increase the distinctiveness of the male identity, which increases the felt pressure to conform to socially accepted expressions of the male gender role.

The hypotheses linking other-gender proportion to gender typicality (H_2b_) and gender discontentedness (H_2d_) were not supported. The null findings warrant further investigation. One possibility is that the datapoints showed a high frequency of being alone (40.5%) and low frequency of being in a predominantly other-gender environment (7.5%), thus reducing variability on the gender salience variable (see Table S1), potentially limiting the observable effects of other-gender proportions. Future studies with more variable data on the proportion of other-gender environments may yield significant effects. It is also worth noting that the infrequency of being in predominantly other-gender groups is an intriguing finding. It illustrates people’s strong tendency to gender-segregate, consistent with research showing spontaneous and persistent same-gender preferences for friends and activities (Leaper, 2022; Mehta & Strough, 2009).

### How does gender identity vary with location?

Location emerged as an important contextual influence on all components of gender identity. At home, participants reported feeling less gender salient (supporting H_3a_), less gender typical (supporting H_3b_), and less pressure to conform to gender stereotypes (supporting H_3c_). These differences may arise when individuals adjust their expectations of their own and others’ behaviours according to expected social roles (Eagly & Wood, 2016; Hyde, 2005). In the private home environment, where there is less social monitoring, people may feel they have more autonomy and freedom of expression than when in public. They may also be less compelled to consider gender as part of their social identity or act in accordance with gender expectations about gender.

Our results showed that although women were generally more discontented with their gender role than men, consistent with previous studies (Cook et al., 2022; Perry et al., 2019), men felt more discontented at home than in other locations. This finding did not support our hypothesis that gender discontentedness would be lower at home where gender is less relevant (H_3d_). One possible interpretation is that the privileges associated with the male identity (Leaper, 1994) may be more readily felt outside the home. For example, in public settings, men are more likely to be selected as leaders than women (Wayne et al., 2010) and men usually experience advantages in terms of position and pay at work compared to women (Lathabhavan & Balasubramanian, 2017). These privileges might be less pronounced in modern homes, potentially leading to greater discontent at home. Future research could further examine the specific factors and experiences that influence men’s discontentedness at home.

### Limitations

Despite its novel contributions, this study has limitations. First, although the repeated and ecologically valid nature of the momentary assessment was an advantage, it increased the risk of boredom and contextual noise. However, the findings replicated many normative findings and were generally consistent with our theoretical predictions, suggesting that the participants contributed thoughtful data. Nevertheless, future studies could include attention check questions and more varied question wording and scale responses to reduce potential response bias.

Second, we focused on gender proportion and home versus other locations as contextual variables. Other variables, like the presence of different companions (e.g., parents vs. strangers), remain to be studied. Although the study design enhanced ecological validity, the nature of our data limited our analytic choices. For example, the small number of data points for predominantly other-gender groups may have reduced the sensitivity of observing the effects of gender proportion, and the low frequencies in locations other than home led us to compare home versus all other locations. We maintain that the ecological method complements and provides unique information that otherwise cannot be demonstrated in laboratory studies. However, future studies could target momentary responses in more specific, selected contexts, such as when participants are at home versus school, or in environments with more extreme gender proportions.

Third, using a Chinese sample provided cross-cultural replication with a diverse sample, but relying on university students may limit generalisability. Nevertheless, we believe that our inclusion of four dimensions of gender identity provided robust internal replication tests for the core research questions, i.e., the extent of variability and how gender identity varies with contexts. Our sample size (*N* = 138 participants, 4,409 reports) was larger than other EMA studies on gender identity (Beltz et al., 2021, *N* = 57, over 4,000 reports; Mehta & Dementieva, 2016, *N* = 27,448 reports). The reliance on young adults was also justifiable as it increased comparability with previous studies.

Fourth, to ensure reasonable completion time, each variable was measured with a few items, as in most EMA studies (Mehta & Dementieva, 2016; Mehta et al., 2016; Solhan et al., 2009; Sunner et al., 2013). Future studies should explore the validity and inclusiveness of these short scales. Due to the need for scale conciseness and the paucity of literature addressing similar questions in gender-diverse samples, the measures asked about only males and females.

Lastly, our study did not intentionally recruit people with other gender identities such as transgender and nonbinary individuals. Despite acknowledging the potential presence of other gender identities, their visibility is low in Chinese societies. Additionally, we aimed to keep the scales brief (e.g., other-gender proportion) for practical reasons. Future research can consider developing scales that examine more diverse gender identities beyond male and female to better reflect people’s experiences.

## Conclusion

To conclude, this study contributes to the theoretical and methodological discourse on gender cognitions by examining temporal variations in gender identity. The results showed clear variations in all gender identity components for most people in everyday life. Two contextual factors, gender proportion and location, were associated with moment-to-moment variations in gender identity. Overall, the findings support the social constructivist view of gender and highlight the dynamic nature of gender identity.

## Supplementary Material

Supplemental Material

## Data Availability

The data that support the findings of this study are available from the corresponding author upon reasonable request.
