# Supplementary material for: Temporal variations in gender identity: an ecological momentary assessment of the influences of context
Source: Aust J Psychol. 2025 Mar 3;77(1):2471056. doi: 10.1080/00049530.2025.2471056 (PMC12218512; doi:10.1080/00049530.2025.2471056)
Supplement: Supplemental Material [file RAUP_A_2471056_SM0750.docx]

**Supplementary Materials**

**Results**

**Additional Analysis**

In our main analyses, other-gender proportion was treated as a continuous variable to preserve variability information. However, this meant that we could not analyze the effects of the participants being alone versus when they were exclusively with same-gender persons separately, as both scenarios were coded as 0. The socialization and social role effects suggest that when alone, people might report lower levels of gender salience, typicality, felt pressure, and discontentedness than when with other people, regardless of the gender composition.

Therefore, we conducted an additional analysis to determine the effect of the participant being alone compared with being with people exclusively of the same or other gender by converting the other-gender proportion into a categorical variable. Because the datapoints showed a high frequency of being alone (40.5%) and low frequency of being in predominantly other-gender environments (7.5%) (Table S1), we recoded the data into the following four categories: 0 = alone, 1 = other-gender proportion smaller than .50 (self as majority gender), 2 = other-gender proportion equal to .50 (own gender equally represented) and 3 = other-gender proportion larger than .50 (self as minority gender).

Next, we ran the same mixed effects models as reported in the main text, except that the other-gender proportion was treated as categorical rather than continuous. The results for the models containing the categorical other-gender variable are reported below and in Table S2. The means are the estimated marginal means adjusted by the model. When the categorical other-gender variable had a significant effect, follow-up analyses were conducted to examine the six possible comparisons: alone vs. other-gender proportion < 0.50 , alone vs. other-gender proportion = 0.50 , alone vs. other-gender proportion > .50, other-gender proportion < .50 vs. other-gender proportion = .50, other-gender proportion < .50 vs. other-gender proportion > .50, and other-gender proportion = .50 vs. other-gender proportion > .50. The results are reported in Table S2.

**Gender Salience.** There was a significant main effect of other-gender (categorical variable), *F*(3, 4294) = 11.01, *p* < .001. There were three significant comparisons among the six possible comparisons: alone vs. other-gender proportion < .50, *F*(1,3511) = 12.29, *p* < .001, *d* = .10, *x̅* _alone_ = 4.25, *x̅* _other-gender proportion < .50_ = 4.38, alone vs. other-gender proportion = .50, *F*(1, 2160) = 22.71, *p* < .001, *d* = .13, *x̅* _alone_ = 4.20, *x̅* _other-gender proportion = .50_ = 4.45, and alone vs. other-gender proportion > .50, *F*(1,2044) = 12.94, *p* < .001, *d* = .25, *x̅* _alone_ = 4.26, *x̅* _other-gender proportion > .50_ = 4.51 (Figure S1a). This pattern of results suggested that gender salience was lower when participants were alone than when with other people, regardless of their gender proportion. The other-gender proportion categories did not significantly differ from each other.

**Gender Typicality.** There was a significant main effect of other-gender (categorical variable), *F*(3, 4289) = 9.88, *p* < .001. There were two significant comparisons among the six possible comparisons: alone vs. other-gender proportion < .50, *F*(1,3505) = 18.19, *p* < .001, *d* = .08, *x̅* _alone_ = 5.06, *x̅* _other-gender proportion < .50_ = 5.16, alone vs. other-gender proportion = .50, *F*(1,2154) = 7.54, *p* = .006, *d* = .05, *x̅* _alone_ = 5.05, *x̅* _other-gender proportion = .50_ = 5.15 (Figure S1b). This pattern of results suggested that gender typicality was lower when participants were alone than when in a group in which their gender was in a majority or with equal gender representation. However, gender typicality was not different when participants were alone and when they were in a group in which their gender was in a minority. The other-gender proportion categories involving multi-person groups did not differ significantly from one another.

**Gender Discontentedness.** There was a significant main effect of other-gender (categorical variable), *F*(3, 4288) = 6.31, *p* < .001, and a significant interaction between gender and other-gender (categorical variable), *F*(3, 4288) = 4.74, *p* = .003. In the follow-up analyses, we examined the six possible comparisons for each gender. For male participants, there were four significant comparisons: alone vs. other-gender proportion < 50%, *F*(1,1654) = 8.08, *p* = .005, *d* = .08, *x̅* _alone_ = 2.49, *x̅* _other-gender proportion < 50%_ = 2.41, alone vs. other-gender proportion > 50%, *F*(1,1146) = 5.11, *p* = .024, *d* = .12, *x̅* _alone_ = 2.49, *x̅* _other-gender proportion < 50%_ = 2.61, other-gender proportion < 50% vs. other-gender proportion > 50%, *F*(1,793) = 12.55, *p* < .001, *d* = .17, *x̅* _other-gender proportion < 50%_ = 2.38, *x̅* _other-gender proportion > 50%_ = 2.55, and other-gender proportion = 50% vs. other-gender proportion > 50%, *F*(1,373) = 9.02, *p* = .003, *d* = .17, *x̅* _other-gender proportion = 50%_ = 2.42, *x̅* _other-gender proportion > 50%_ = 2.61 (Figure S1c(i)). This pattern of results largely aligned with the distinctiveness effects predicted by DIT and distinctiveness theory, because male participants were more discontented when they were in a male minority group than when they were either alone or in a male majority group. However, the effect of being observed was inconsistently supported, because men were more discontented when alone than when they were in a male majority group, but they were less discontented when they were in a male minority group. For females, there was one significant comparison, alone vs. other-gender proportion = .50, *F*(1,922) = 6.83, *p* = .009, *d* = .14, *x̅* _alone_ = 2.85, *x̅* _other-gender proportion = .50_ = 2.71 Figure S1c(ii)). Women were more discontented when alone than when equally represented in groups, which did not support the hypothesized effects.

**Felt Pressure to Conform to Gender Stereotypes.** There was a significant main effect of other-gender (categorical variable), *F*(3, 4291) = 10.74, *p* < .001. In the follow-up analyses, there were three significant comparisons among the six possible comparisons: alone vs. other-gender proportion < 50%, *F*(1,3507) = 23.18, *p* < .001, *d* = .10, *x̅* _alone_ = 3.61, *x̅* _other-gender proportion < 50%_ = 3.77, alone vs. other-gender proportion = 50%, *F*(1,2156) = 8.56, *p* = .003, *d* = .02, *x̅* _alone_ = 3.59, *x̅* _other-gender proportion = 50%_ = 3.75, and alone vs. other-gender proportion > 50%, *F*(1,2036) = 7.68, *p* = .006, *d* = .10, *x̅* _alone_ = 3.62, *x̅* _other-gender proportion > 50%_ = 3.81 (Figure S1d). This pattern of results suggested that felt pressure was lower when participants were alone than when with other people, regardless of their gender proportion. The other gender proportion categories did not significantly differ from each other.

**Discussion**

**Effects of Being Alone**

The main focus of this additional analysis was to separate the effect of being alone from the effect of being with others of different gender compositions by making the gender proportion variable categorical. In the main analysis, when the other-gender proportion increased, both men and women reported higher gender salience, and men reported higher pressure to conform to gender stereotypes. In this additional analysis, compared with being alone, participants reported higher gender salience, typicality, and pressure to conform to gender stereotypes when with others, regardless or largely regardless of the group’s gender proportion. These findings are consistent with the established findings showing that gender differences tend to be larger when participants are aware of being observed, aligned with socialization and social role theories (Hyde, 2005; Leaper & Friedman, 2007). In other words, the presence of others, regardless of their gender, may increase attention to gender and impose gender socialization pressure on individuals.

**Effects of Gender Proportion**

The effect of gender proportion was minimal in this additional analysis. When “being alone” was singled out, only male participants’ gender discontentedness showed some support for the gender proportion effect, as it was higher when the male participants were in a male minority group than when they were in a male majority group or alone. The reduced support for the gender proportion effect is likely due to the loss of data variability when converting the other-gender proportion from a continuous variable to a categorical variable.

The main analysis with other-gender proportion as a continuous variable may have been a more meaningful and feasible choice of analysis for several reasons. First, although considering other-gender proportion as a continuous variable meant that we could not differentiate between being alone and being in a same-gender group, the purpose of differentiating between these two scenarios was that they differ in terms of privacy and degree of social monitoring. To this end, the main analysis included location in the model, which meant that the extent of privacy and social monitoring was controlled to some extent when the effect of gender proportion was being tested. Second, as shown by the frequency distribution of the other-gender proportion categories (Table S1), there were insufficient data points where the other-gender proportion was equal to or larger than .50 (representing 10.6% and 7.5% of the data, respectively). This limited the number of categories that could be created to represent the different gender proportion groups and also the sensitivity of the categorical analysis. Third, excluding cases where the participants were alone in the main analysis while keeping gender proportion as a continuous variable may not be a good option because this would entail the removal of half of the data points. This reduction in sample size would limit the power of the analysis for testing both gender proportion and location. Fourth, we acknowledge that our main analyses could be improved by controlling for the number of people present, i.e., group size, but in real world settings, it might be difficult for participants to estimate the exact number of men and women (e.g., in crowded environments) and introduce more “noise” compared with only estimating the proportions.

| Table S1  *Frequency Table of Different Gender Proportion Categories* | |
| --- | --- |
| Gender Proportion Categories | Frequency |
| When the participant is alone | 40.5% |
| When the other-gender proportion is < .50 | 41.5% |
| When the other-gender proportion is = .50 | 10.6% |
| When the other-gender proportion is > .50 | 7.5% |

| Table S2  *Results of General Linear Mixed Effects Models (Gender Proportion as a Categorical Variable)* | | | | | |
| --- | --- | --- | --- | --- | --- |
|  | Main Effects | | | Interactions | |
| Gender Identity Variables | **Other-gender (categorical variable)** | Location | Gender | **Gender × Other-gender (categorical variable)** | Gender × Location |
| Gender Salience | ***F*(3, 4294) = 11.01, *p* < .001** | *F*(1, 4298) = 54.63, *p* < .001 | *F*(1, 141) = 7.82, *p* = .006 | *F*(3, 4294) = 2.29, *n.s.* | *F*(1, 4298) = .47, *n.s.* |
| Gender Typicality | ***F*(3, 4289) = 9.88, *p* < .001** | *F*(1, 4292) = 12.69, *p* < .001 | *F*(1, 140) = 9.77, *p* =.002 | *F*(3, 4289) = .53, *n.s.* | *F*(1, 4292) = .03, *n.s.* |
| Gender Discontentedness | ***F*(3, 4288) = 6.31, *p* < .001** | *F*(1, 4291) = .10, *n.s.* | *F*(1, 140) = 8.75, *p* = .004 | ***F*(3, 4288) = 4.74, *p* = .003** | *F*(1, 4291) = 3.09, *n.s.* |
| Felt Pressure to Conform to Gender Stereotypes | ***F*(3, 4291) = 10.74, *p* < .001** | *F*(1, 4294) = 56.78, *p* < .001 | *F*(1, 141) = 14.37, *p* < .001 | *F*(3, 4290) = 2.56, *n.s.* | *F*(1, 4294) =.006, *n.s.* |

| S1a | 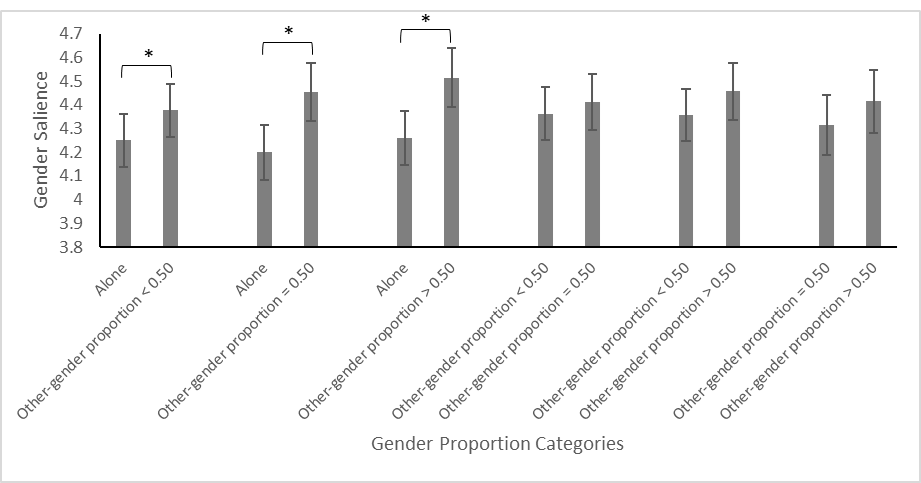 |
| --- | --- |
| S1b | 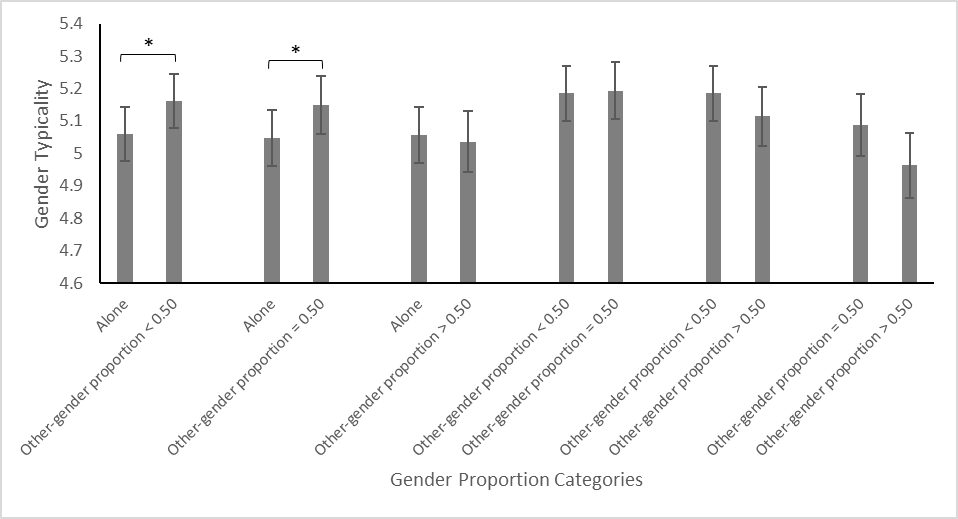 |
| S1c(i) | 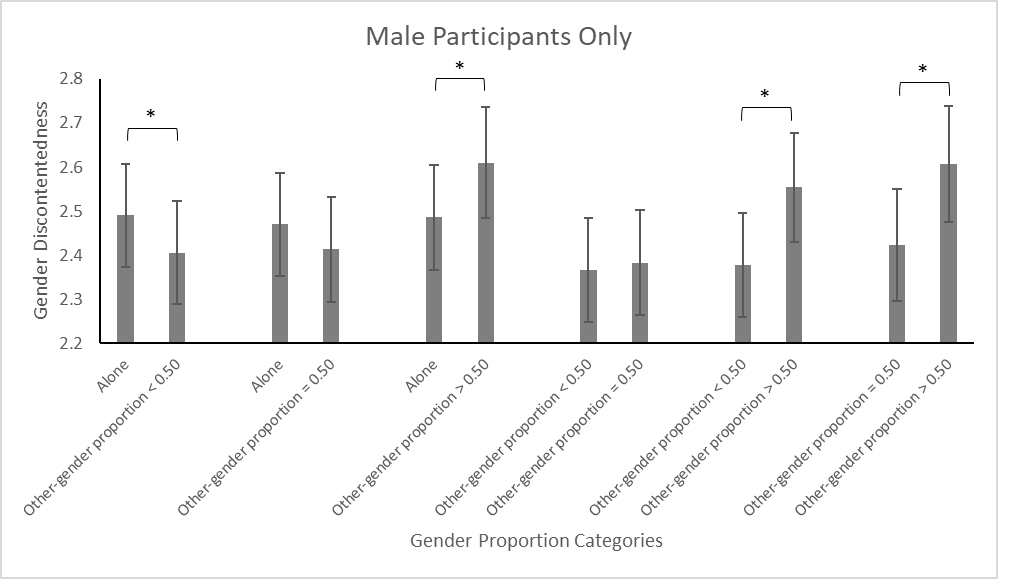 |
| S1c(ii) | 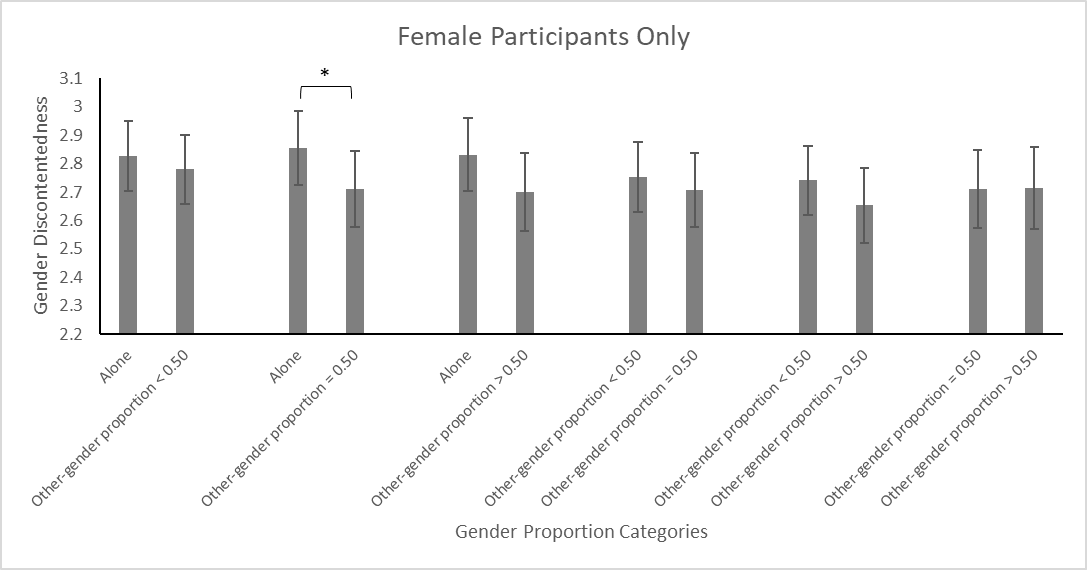 |
| S1d | 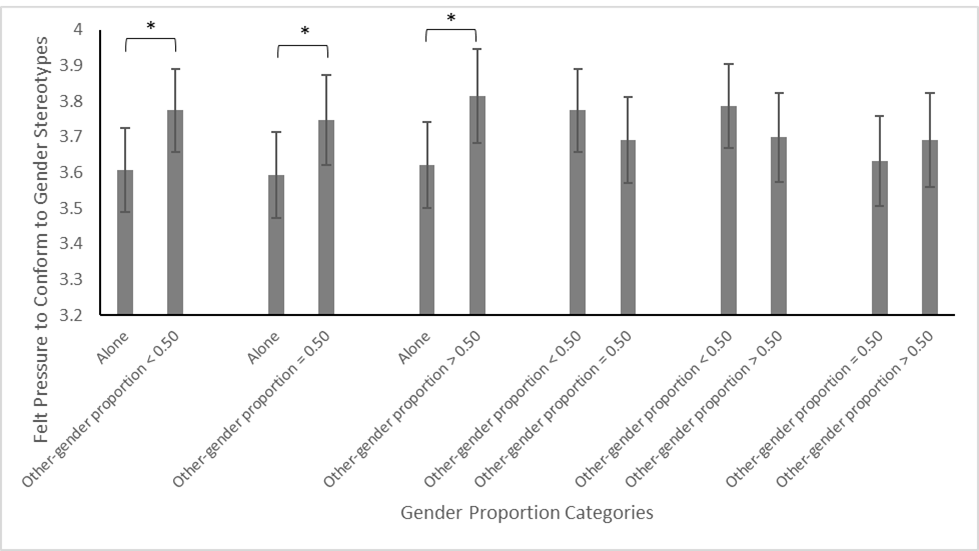 |
| *Figure S1.* Main effects of the presence of other-gender for each gender identity component. S1a: Gender Salience; S1b: Gender Typicality; S1c(i): Gender Discontentedness for male participants; S1c(ii): Gender Discontentedness for female participants; S1d: Felt Pressure to Conform to Gender Stereotypes | |

References

Hyde, J. S. (2005). The gender similarities hypothesis. *American Psychologist*, *60*(6), 581-592. <https://doi.org/10.1037/0003-066X.60.6.581>

Leaper, C., & Friedman, C. K. (2007). The socialization of gender. In J. E. Grusec & P. D. Hastings (Eds.), *Handbook of socialization: Theory and research* (pp. 561-587). New York: Guilford.
